# Differential diagnostic value of tumor markers and contrast-enhanced computed tomography in gastric hepatoid adenocarcinoma and gastric adenocarcinoma

**DOI:** 10.3389/fonc.2023.1222853

**Published:** 2023-07-19

**Authors:** Congsong Dong, Yanling Wang, Xiaoyu Gu, Xiaojing Lv, Shuai Ren, Zhongqiu Wang, Zhenyu Dai

**Affiliations:** ^1^Department of Radiology, Affiliated Hospital 6 of Nantong University (Yancheng Third People’s Hospital), Yancheng, China; ^2^Department of Radiology, The People’s Hospital of Suzhou New District, Suzhou, China; ^3^Department of Radiology, Affiliated Hospital of Nanjing University of Chinese Medicine, Nanjing, China

**Keywords:** gastric cancer, hepatoid adenocarcinoma, α-fetoprotein (AFP), computed tomography, differential diagnosis

## Abstract

**Objective:**

This study aimed to investigate the effectiveness of tumor markers and contrast-enhanced computed tomography (CE-CT) in differentiating gastric hepatoid adenocarcinoma (GHA) from gastric adenocarcinoma (GA).

**Methods:**

This retrospective study included 160 patients (44 with GHA vs. 116 with GA) who underwent preoperative CE-CT. Preoperative serum concentrations of tumor biomarkers and CT imaging features were analyzed, including alpha-fetoprotein (AFP), carcinoembryonic antigen (CEA), carbohydrate antigen 19-9 (CA19-9), carbohydrate antigen 125 (CA125), tumor location, growth pattern, size, enhancement pattern, cystic changes, and mass contrast enhancement. Multivariate logistic regression analyses were performed to evaluate useful tumor markers and CT imaging features for differentiating GHA from GA.

**Results:**

When compared to GA, GHA showed a higher serum AFP [13.27 ng/ml (5.2–340.1) vs. 2.7 ng/ml (2.2–3.98), *P <*0.001] and CEA levels [4.07 ng/ml (2.73–12.53) vs. 2.42 ng/ml (1.38–4.31), *P <*0.001]. CT imaging showed GHA with a higher frequency of tumor location in the gastric antrum (*P <*0.001). GHA had significantly lower attenuation values at the portal venous phase [PCA, (82.34 HU ± 8.46 vs. 91.02 HU ± 10.62, *P <*0.001)] and delayed phase [DCA, (72.89 HU ± 8.83 vs. 78.27 HU ± 9.51, *P <*0.001)] when compared with GA. Multivariate logistic regression analyses revealed that tumor location, PCA, and serum AFP level were independent predictors of differentiation between GHA and GA. The combination of these three predictors performed well in discriminating GHA from GA, with an AUC of 0.903, a sensitivity of 86.36%, and a specificity of 81.90%.

**Conclusions:**

Integrated evaluation of tumor markers and CT features, including tumor location, PCA, and serum AFP, allowed for more accurate differentiation of GHA from GA.

## Introduction

Hepatoid adenocarcinoma (HA) is a special type of extrahepatic adenocarcinoma that resembles hepatocellular carcinoma (HCC) owing to its morphological and immunohistochemical properties, and HA is characterized by a poor prognosis (1). The estimated annual incidence of HA is 0.58–0.83 cases per million individuals (2). HA has been reported to occur in many organs, including the pancreas, esophagus, lung, gallbladder, colon, ovaries, uterus, peritoneum, and other sites, but mostly in the stomach (3–9).

The term ‘hepatoid adenocarcinoma of the stomach’ was first proposed by Ishikura et al. in 1986. Gastric hepatoid adenocarcinoma (GHA) is histologically characterized by hepatoid differentiation and the production of large amounts of alpha-fetoprotein (AFP) (10). GHA always exhibits early lymphatic and hepatic metastases and is associated with a poor prognosis compared with gastric adenocarcinoma (GA) (11). According to current research, the management strategy for GHA is similar to that for GA (12). Radical surgery and adjuvant therapy are the standard treatments for resectable GHA (12, 13). However, early disease recurrence and poor prognosis are still observed in GHA despite radical surgery with free margins (14). Zhou et al. (11) recommended a combination treatment of radical gastrectomy and sufficient adjuvant chemotherapy for patients with GHA considering that it yielded better survival. Therefore, preoperative differentiation of GHA from GA is of great clinical significance as it facilitates strategic management and prognosis prediction.

The majority of patients with GHA patients showed elevated AFP concentration, which was positively associated with the hepatoid adenocarcinoma cell component (15). However, it is noted that there were still patients with GHA whose serum AFP levels were negative despite being pathologically confirmed (16). It has been also reported that other hematological markers, such as the concentrations of serum carbohydrate antigen 19-9 (CA19-9), carbohydrate antigen 125 (CA125), and carcinoembryonic antigen (CEA), were also elevated in some GHA cases (2).

Endoscopic ultrasound-guided fine-needle aspiration (EUS-FNA) is commonly performed in routine clinical practice. However, biopsy samples may not accurately reflect the entire extent of the phenotype of the whole tumor. Computed tomography (CT) has become an important noninvasive method for the diagnosis and evaluation of focal solid masses. CT shows great promise in GHA diagnosis since it allows the evaluation of metastatic lymph nodes, which is a significant predictor for differentiating GHA from other gastric cancers (17, 18). Additionally, patterns of enhancement on contrast-enhanced CT (CE-CT) may also provide information for diagnosis, since hypo-enhancement at the arterial phase was more frequently observed in GHA than in HCC (2). This study aimed to evaluate the diagnostic value of tumor markers and CE-CT in differentiating between GHA and GA.

## Materials and methods

### Patients

**Figure 1:**
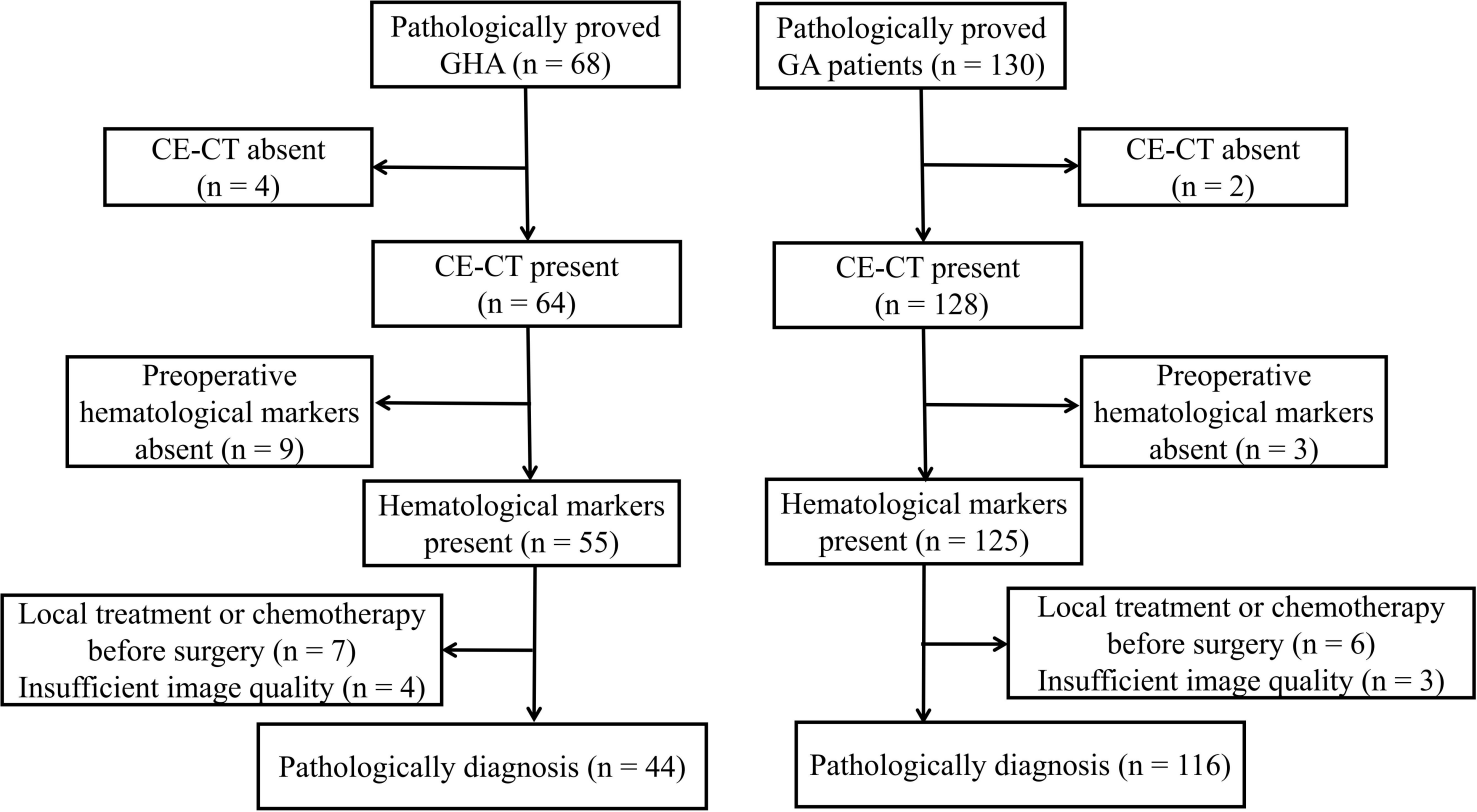
Flowchart of patients throughout the study.

This study was approved by our ethics committee, and the requirement for informed consent was waived owing to its retrospective nature. We searched our radiology database for patients with GHA between January 2013 and January 2021. To create a suitable comparison group, we searched for GA cases between January 2018 and January 2021. A total of 198 patients were identified in the database search (68 GHA and 130 GA). The following inclusion criteria were used: a) pathologically proven GHA or GA with surgery; b) patients who underwent preoperative CT within 30 days prior to surgery; c) preoperative hematological markers were available, including AFP, CEA, CA19-9, and CA125; d) patients without any prior history of cancer; and e) patients who did not receive local treatment or systemic chemotherapy before surgery. According to the inclusion criteria, 24 patients with GHA were excluded from the study, i.e., four patients without available CE-CT, nine patients without preoperative hematological markers, seven patients received local treatment or systemic chemotherapy before surgery, and four patients with insufficient image quality. Finally, 44 patients with GHA (36 men and eight women; mean age, 64.32 years ± 7.40 [SD]) were included in the study (**Figure 1**). Fourteen patients with GA were excluded from the study i.e., two patients without available CE-CT, three patients without preoperative hematological markers, six patients received local treatment or systemic chemotherapy before surgery, and three patients with insufficient image quality. Finally, 116 patients with GA (77 men and 39 women; mean age, 64.84 years ± 9.95 [SD]) were included in the study (**Figure 1**). Preoperative serum markers, including AFP, CEA, CA19-9, and CA125, were recorded for further analysis.

### CT examinations

Unenhanced CT and triple-phase enhanced CT (arterial, portal venous, and delayed phases) were performed prior to surgery using a 64-section helical CT scanner (Discovery HD750, GE Healthcare, Milwaukee, Wisconsin, USA; Light speed, VCT, GE Healthcare, Milwaukee, WI; Philips Brilliance 64, Philips Healthcare, DA Best, the Netherlands). To better visualize the gastric wall, all patients fasted for 8 h and had 1,000 mL of water 15 min before the CT scan. Following CT scanning parameters were used: 120-kV tube voltage; 250–400 mA tube current; 0.75–1.0 pitch; 0.5–0.75 s rotation time; 3.0–5.0 mm section thickness. The arterial, portal venous, and delayed phases were acquired at 25-second, 60-second, and 120-second delay after intravenous administration of nonionic contrast material (1.5 ml/kg) at a rate of 3.0 ml/s. The original images were reconstructed with a 2-mm section thickness and 2-mm section interval. Multiplanar reformatting was performed to better visualize the lesions when necessary.

### Image analysis

Image analysis was performed with reference to the axial images; however, sagittal and coronal images were also referenced when necessary. Two abdominal radiologists, who were blinded to the pathological results, independently reviewed the CT images. In cases with discrepancies, consensus was reached by referral to a third radiologist with 11 years of experience in abdominal CT reading. The following imaging features were assessed: tumor location (cardia and body vs. antrum), growth pattern (infiltrative growth, ulceration, and polypoid), tumor size, tumor contrast enhancement pattern (homogeneous vs. heterogeneous), cystic changes, and enlarged lymph node. Infiltrative growth was defined as an unclear border between the lesion and normal gastric wall (19). Ulceration was defined as a local depression at the center or on the surface of the lesion (19). Polypoids were defined as lobulated or fungating masses with or without ulceration. Cystic changes were defined as non-enhancing oval or circular shapes with well-defined margins (20). Enlarged lymph nodes were defined as lymph nodes ≥10 mm in the short-axis dimension of the upper abdomen (19).

CT attenuation values of all lesions in the unenhanced, arterial, portal venous, and delayed phases were measured in Hounsfield units (HUs) using a 100-mm^2^ circular region of interest (ROI). The ROI was carefully placed to include as much of the most strongly enhanced area of the lesions as possible, and to avoid necrotic or adjacent structures (17). Each lesion was measured three times, and the mean values were calculated and recorded (17).

### Statistical analysis

Statistical analysis was performed using SPSS 26.0 (IBM Corporation, Armonk, NY, USA). Categorical variables are presented as numbers (percentages) and compared using the χ^2^ test or Fisher’s exact test. Continuous variables are presented as mean ± standard deviation (SD) and compared using either Student’s t-test or the Mann–Whitney U test. The Shapiro–Wilk test was used to test the normality of the continuous variables. Discrete variables are presented as medians (25% quartile, 75% quartile) and using the Kruskal–Wallis test. A binary logistic regression model was established to identify independent predictors, and receiver operating characteristic (ROC) curves were used to evaluate the diagnostic performance of tumor markers and CT features in discriminating GHA from GA. The DeLong test was used to compare the statistical significance of ROC curves. Time-dependent density curves (TIDs) of GHA and GA were obtained using 64-bit OriginPro 8.6 using the CT attenuation values of all lesions. Statistical significance was set at *P*-value <0.05.

## Results

### Clinical characteristics and tumor markers

Patient clinical characteristics and tumor markers are shown in **Table 1**. In this study, 44 patients with GHA (36 men and eight women; mean age, 64.32 years ± 7.40 [SD]) and 116 patients with GA (77 men and 39 women; mean age, 64.84 years ± 9.95 [SD]) were finally included. No significant differences were found in the age or sex distribution between GHA and GA. For tumor markers, no significant differences were found in CA 19-9 [8.18 U/ml (4.02–14.51) vs. 7.53 U/ml (3.13–19.40), *P* = 0.751] or CA125 levels [8.90 U/ml (5.96–13.66) vs. 10.25 U/ml (7.0–15.03), *P* = 0.751] between GHA and GA. Patients with GHA showed higher serum AFP [13.27 ng/ml (5.2–340.1) vs. 2.7 ng/ml (2.2–3.98), *P <*0.001] and CEA levels [4.07 ng/ml (2.73–12.53) vs. 2.42 ng/ml (1.38–4.31), *P <*0.001] as compared to those of patients with GA.

**Table 1:** Clinical characteristics in patients with gastric hepatoid adenocarcinoma (GHA) and gastric adenocarcinoma (GA).

Parameters	GHA (n = 44)	GA (n = 116)	*P*-value
Gender			0.056
Male	36 (81.8%)	77 (66.4%)	
Female	8 (18.2%)	39 (33.6%)	
Age (years)	64.3 ± 7.4	64.8 ± 9.9	0.468
^*^Serum AFP (ng/ml)	13.27 (5.20–340.10)	2.7 (2.20–3.98)	<0.001
^*^Serum CEA (ng/ml)	4.07 (2.73–12.53)	2.42 (1.38–4.31)	<0.001
^*^Serum CA19-9 (U/ml)	8.18 (4.02–14.51)	7.53 (3.13–19.40)	0.751
^*^Serum CA125 (U/ml)	8.90 (5.96–13.66)	10.25 (7.00–15.03)	0.178

^*^ indicates medians (25% quartile–75% quartile); AFP, alpha-fetoprotein; CEA, carcinoembryonic antigen; CA19-9, carbohydrate antigen 19-9; CA125, carbohydrate antigen 125.

### CT image analysis

**Figure 2:**
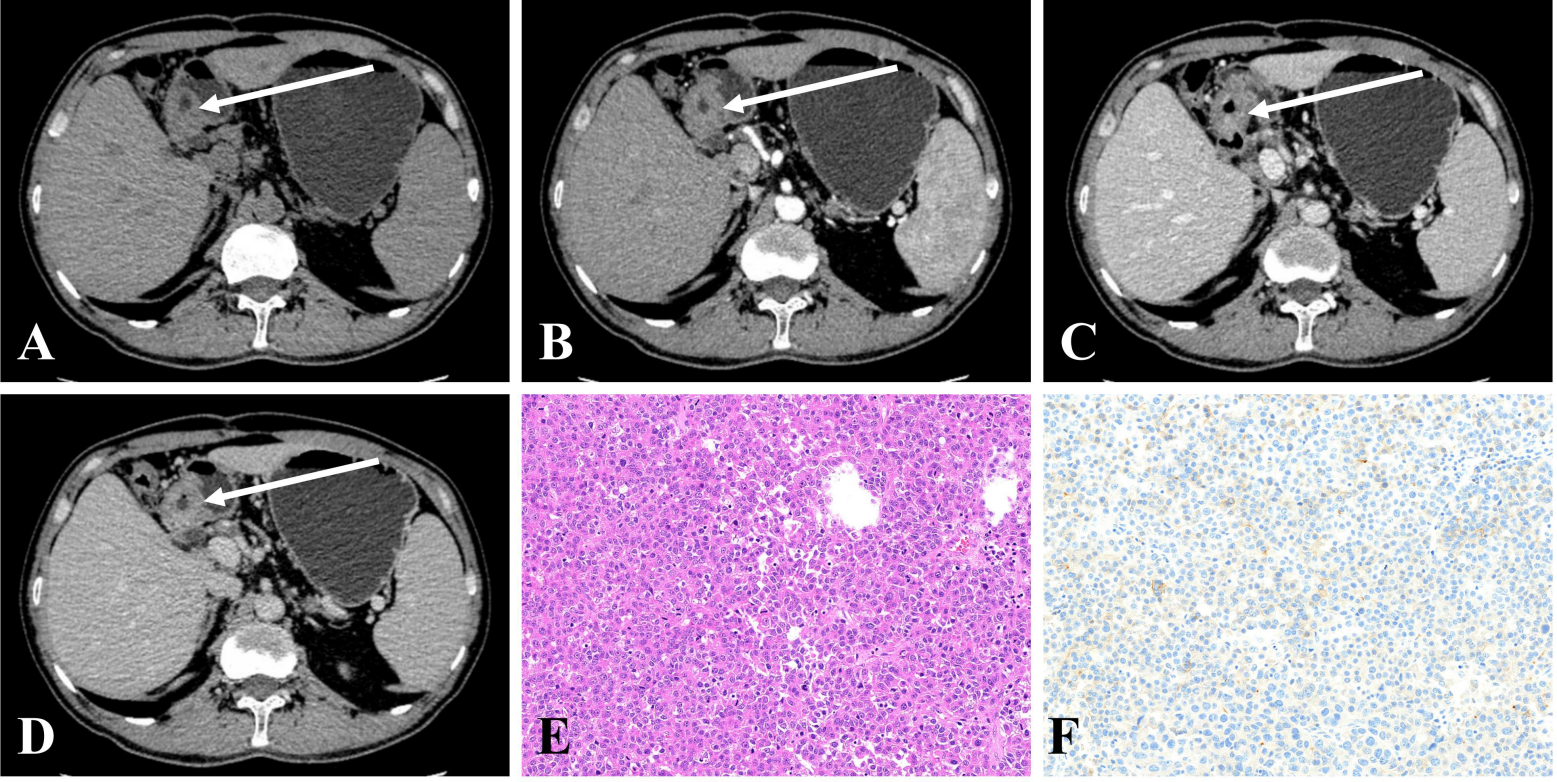
**(A–D)** CE-CT in a 50-year-old man pathologically proven to have GHA with high serum AFP (100.7 ng/mL) showed an irregular thickened wall in the gastric antrum with heterogeneous enhancement. The CT attenuation values of the lesions were 43, 57, 76, and 69 HU in the unenhanced, arterial, portal venous, and delayed phases, respectively. **(E)** Microscopic examination (hematoxylin and eosin stain, ×200) revealed poorly differentiated regions mimicking hepatocellular carcinoma. **(F)** Postoperative immunohistochemical analysis (Her-2 staining, ×200) showing that Her-2 expression was 1+.

**Figure 3:**
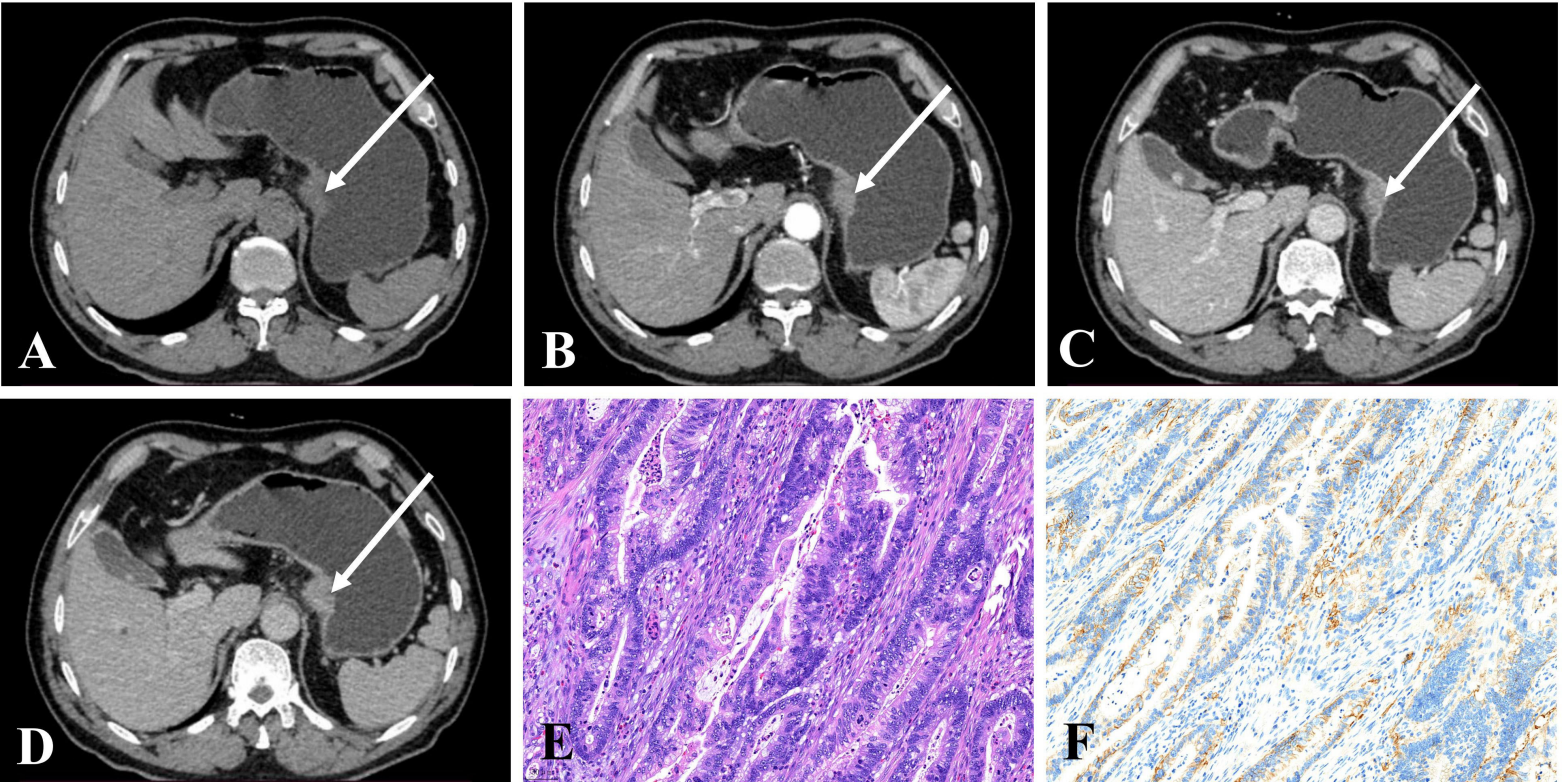
**(A–D)** CE-CT of a 68-year-old man pathologically proven to have GA with normal serum AFP (1.9 ng/mL) showed a nodular thickened wall in the gastric cardia with homogeneous enhancement. The CT attenuation values of the lesions were 43, 74, 91, and 78 HU in the unenhanced, arterial, portal venous, and delayed phases, respectively. **(E)** Microscopic examination (hematoxylin and eosin stain, ×200) showing poorly and moderately differentiated tubular adenocarcinoma. **(F)** Postoperative immunohistochemical analysis (Her-2 staining, ×200) showed that Her-2 expression was 2+.

The results of the qualitative CT imaging of GHA and GA are shown in **Table 2**. There were 29 cases out of 44 (65.9%) patients had lesions in the gastric antrum and 15 in the gastric cardia and body (34.1%) in the GHA group. There were 40 cases out of 116 (34.5%) had lesions in the gastric antrum and 76 in the gastric cardia and body (65.5%) for GA group. The tumor location of the lesions was significantly different between the two groups (*P <*0.001). The most common growth pattern for both GHA and GA was ulcerative (81.8% vs. 77.6%). Both tumors showed a high frequency of heterogeneous enhancement patterns (86.4% vs. 82.8%) and a low frequency of cystic changes (9.1% vs. 6.0%). Both the GHA and GA groups showed a high frequency of enlarged lymph nodes (86.4% vs. 79.3%). There were no significant differences between these two entities regarding the growth pattern, enhancement pattern, cystic changes, or enlarged lymph nodes. Representative cases of GHA and GA are shown in **Figures 2**, **3**.

**Table 2:** CT imaging findings in patients with gastric hepatoid adenocarcinoma (GHA) and gastric adenocarcinoma (GA).

Parameters	GHA (n = 44)	GA (n = 116)	*P*-value
Tumor location			<0.001
Cardia & body	15 (34.1%)	76 (65.5%)	
Antrum	29 (65.9%)	40 (34.5%)	
Growth pattern			0.796
Polypoid	5 (11.4%)	18 (15.5%)	
Ulceration	36 (81.8%)	90 (77.6%)	
Infiltrative growth	3 (6.8%)	8 (6.9%)	
Tumor enhancement pattern			0.581
Homogeneous	6 (13.6%)	20 (17.2%)	
Heterogeneous	38 (86.4%)	96 (82.8%)	
Cystic changes			0.495
Present	4 (9.1%)	7 (6.0%)	
Absent	40 (90.9%)	109 (94.0%)	
Tumor size (cm)	4.39 ± 1.65	3.94 ± 1.59	0.067
CT attenuation values (HU)			
Unenhanced phase	37.93 ± 3.27	39.03 ± 3.77	0.083
Arterial phase	64.50 ± 7.26	66.95 ± 8.19	0.084
Portal venous phase	82.34 ± 8.46	91.02 ± 10.62	<0.001
Delayed phase	72.89 ± 8.83	78.27 ± 9.51	<0.001

cm, centimeter; HU, Hounsfield units.

**Figure 4:**
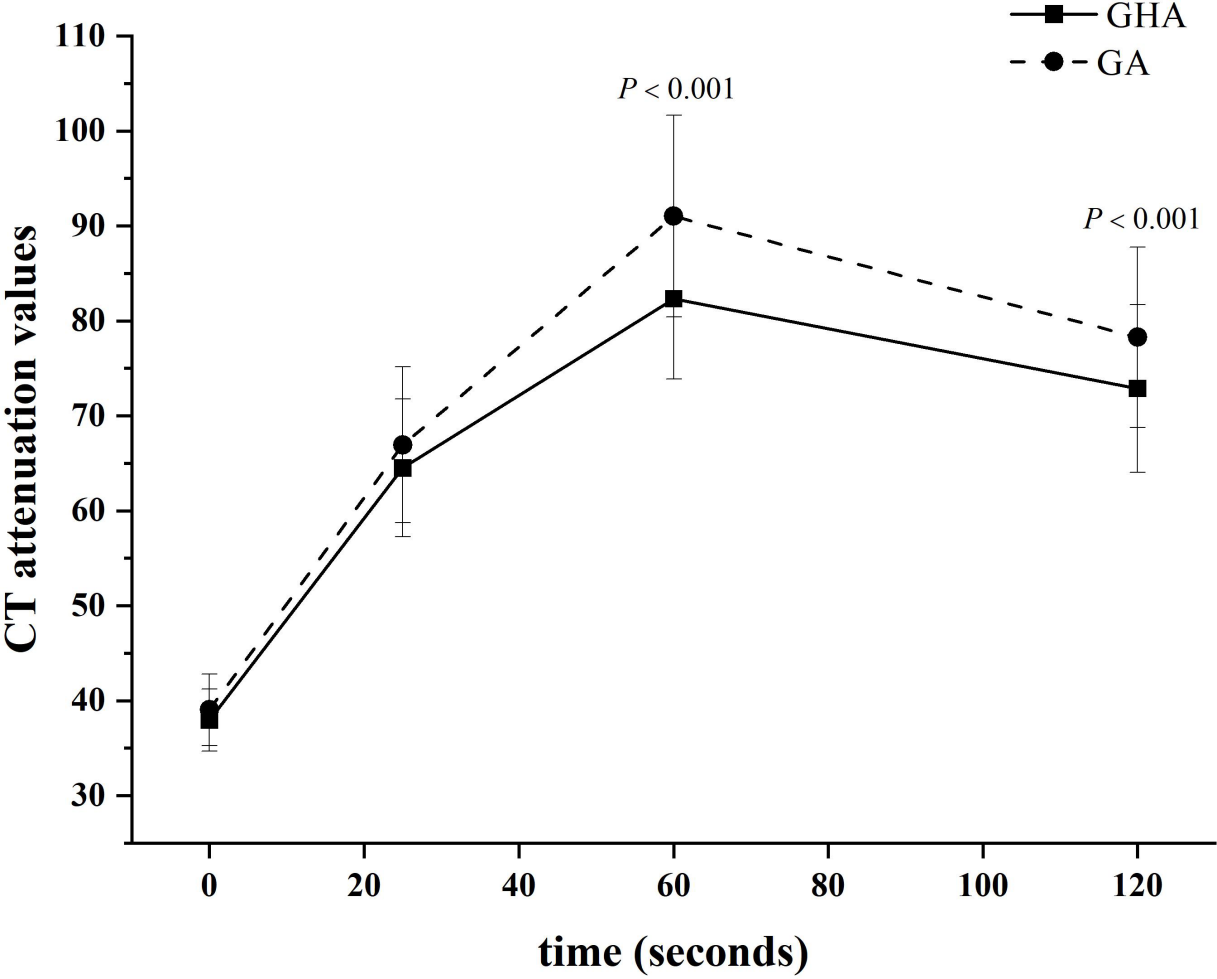
Time-dependent density curves for GHA and GA. The CT attenuation values of GHA and GA were 37.93 ± 3.27 and 39.03 ± 3.77 Hounsfield units in unenhanced CT images, respectively. The CT attenuation values of GHA were significantly lower than those of GA in the portal venous and delayed phases (both *P <*0.001).

The results of the quantitative CT imaging of GHA and GA are shown in **Table 2**. The attenuation values of GHA at portal venous phase (82.34 HU ± 8.46 vs 91.02 HU ± 10.62, *P <*0.001) and delayed phase (72.89 HU ± 8.83 vs 78.27 HU ± 9.51, *P <*0.001) were significantly lower than those of GA, while there was no significant difference in unenhanced (37.93 HU ± 3.27 vs 39.03 HU ± 3.78, *P* = 0.083) or arterial (64.50 HU ± 7.26 vs 66.95 HU ± 8.19, *P* = 0.084) CT attenuation values between GHA and GA (**Figure 4**).

### Diagnostic performance of tumor markers and CT features in discriminating GHA from GA

Binary logistic regression analysis was used to analyze the variables [AFP, CEA, tumor location, CT attenuation values at the portal venous phase (PCA), and delayed phase (DCA)] with significant between-group differences. Binary logistic regression analysis revealed that tumor location, PCA, and FAP were independent predictors for discriminating GHA from GA (**Table 3**).

**Table 3:** Binary logistic regression analysis of qualitative and quantitative CT features in differentiating GHA from GA.

Parameters	Odds ratio (95% CI)	Wald	*P*-value
Tumor location	3.441 (1.256–9.429)	5.774	0.016
PCA	0.885 (0.806–0.973)	6.456	0.011
DCA	1.029 (0.944–1.121)	0.42	0.517
Serum AFP	1.113 (1.026–1.207)	6.592	0.01
Serum CEA	1.001 (0.996–1.006)	0.084	0.772

PCA, CT attenuation value at portal venous phase; DCA, CT attenuation value at delayed phase; AFP, alpha-fetoprotein; CEA, carcinoembryonic antigen.

**Figure 5:**
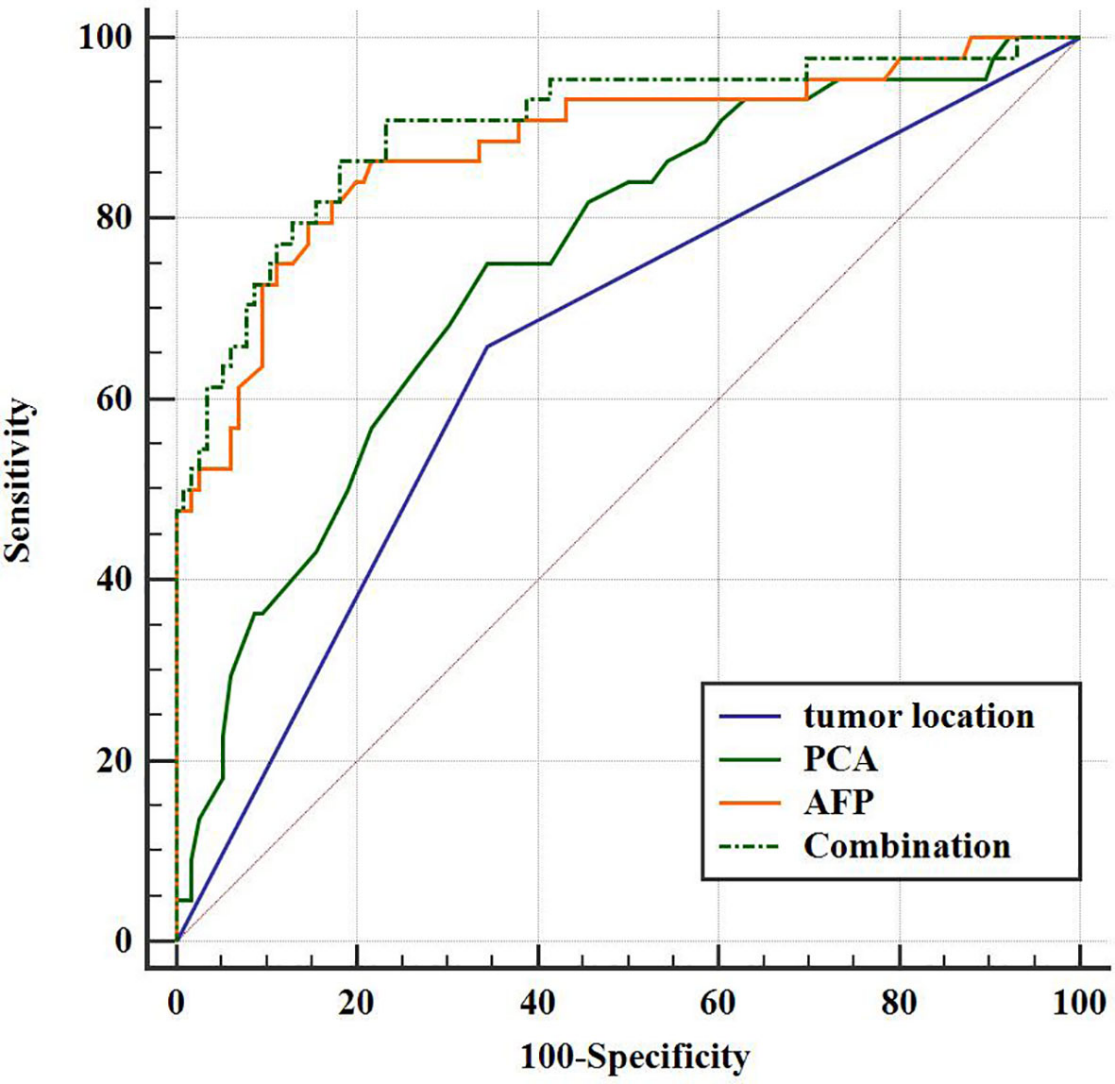
Receiver operating characteristic (ROC) curve for the diagnostic performance of tumor location, PCA, AFP, and their combinations regarding the differentiation between GHA and GA.

The diagnostic performances of tumor location, AFP, PCA, and their combinations for discriminating GHA from GA are summarized in **Table 4**. Using ROC curves, we determined the cut-off value of AFP as 4.7 ng/ml with 79.6% sensitivity and 85.3% specificity with an area under the curve (AUC) of 0.881. Similarly, we determined the cutoff value of PCA as 85 HU with 75.0% sensitivity and 65.5% specificity with an AUC of 0.749 (**Figure 5**). The combination of these three variables (tumor location, AFP, and PCA) yielded 86.4% sensitivity and 81.9% specificity, with an AUC of 0.903 (**Figure 5**). The DeLong test showed that there were statistically significant differences in the AUC among tumor location, PCA, and their combinations (tumor location vs. combination, *P <*0.001; PCA vs. combination, *P <*0.001). However, there was no statistically significant difference in the AUC between AFP and the combination therapy (*P =* 0.5786).

**Table 4:** Diagnostic performance of CT features in differentiating GHA from GA.

Parameters	AUC	95% CI	Sensitivity	Specificity	Youden index
Tumor location	0.657	0.578–0.730	65.91%	65.52%	0.3143
PCA	0.749	0.675–0.814	75.00%	65.52%	0.4052
Serum AFP	0.881	0.820–0.927	79.55%	85.34%	0.6489
Combination	0.903	0.846–0.944	86.36%	81.90%	0.6826

AUC, area under curve; CI, confidence interval; PCA, CT attenuation value at portal venous phase; AFP, alpha-fetoprotein.

## Discussion

GHA and GA share similar clinical symptoms and imaging findings (16, 17). However, GHA always exhibits early lymphatic and hepatic metastases and is associated with a poorer prognosis than GA (11). Elevated AFP is observed in most patients with GHA, but normal AFP levels have also been reported (21). Additionally, some GA patients also had elevated AFP levels, which is called AFP-producing gastric cancer (9). Therefore, more reliable biomarkers with high pooled sensitivity and specificity for the discrimination of GHA from GA are urgently required. Our study results demonstrated that AFP, PCA, and tumor location are statistically significant predictors in the differential diagnosis of GHA from GA. The combination of AFP, PCA, and tumor location had a good diagnostic performance in the discrimination of GHA from GA with 86.4% sensitivity, 81.9% specificity and an AUC of 0.903.

Clinically, GHA mostly occurs in elderly male individuals without specific clinical characteristics and imaging findings (11, 15). The average age of patients with GHA is 62.45 years, ranging from 56 to 74 years (17). In patients with GHA, the male-to-female ratio was 3.2 to 1 (22). In our study, the mean age of patients is 64.3 years, ranging from 44 to 81 years. The male-to-female ratio was 4.5 to 1 (36 male vs. eight female).

Approximately 80% of patients with GHA demonstrate elevated serum AFP levels, which can also be seen in patients with HCC, cirrhosis, and hepatitis; however, normal serum levels have also been reported in patients with GHA (16). In our study, the positivity rate of high serum AFP in patients with GHA was 52.3% (23/44 patients). Patients with GHA showed a higher AFP level than patients with GA [13.27 ng/ml (5.2–340.1) vs. 2.7 ng/ml (2.2–3.98), *P <*0.001]. Lin et al. found that a preoperative CEA level ≥5 ng/mL was independently associated with GHA and that the prognosis of patients with elevated CEA levels was significantly worse than that of patients with normal CEA levels; therefore, clinical attention to CEA levels is warranted (22). In our study, the positive rate of high serum CEA levels in patients with GHA was 38.6% (17/44 patients). Patients with GHA showed higher CEA levels than those patients with GA [4.07 ng/ml (2.73–12.53) vs. 2.42 ng/ml (1.38–4.31), *P <*0.001]. It has also been reported that the serum concentrations of CA19-9 and CA125 are elevated in some GHA cases (2). In our series, the positivity rates of high serum CA19-9 and CA125 levels in patients with GHA were 2.27% (1/44 patients) and 38.6% (17/44 patients), respectively. No significant differences were found in CA 19-9 [8.18 U/ml (4.02–14.51) vs. 7.53 U/ml (3.13–19.40), *P* = 0.751] or CA125 levels [8.90 U/ml (5.96–13.66) vs. 10.25 U/ml (7.0–15.03), *P* = 0.751] between GHA and GA.

CT has been proved to be a useful tool for diagnosing GHA (1, 17). Fu et al. revealed that lesions in the arterial phase minus the portal venous phase and the lesion/aorta ratio were statistically significant predictors of differentiation of GHA from GA (17). In our study, we found that tumor location and CT attenuation values in the portal venous phase (PCA) and delayed phase (DCA) were statistically significantly different between GHA and GA. Patients with GHA showed a higher frequency of lesions in the gastric antrum than those with GA [29/44 (65.9%) vs. 40/116 (34.5%), *P <*0.001]. Additionally, GHA had a lower PCA (82.34 HU ± 8.46 vs 91.02 HU ± 10.62) and DCA (72.89 HU ± 8.83 vs 78.27 HU ± 9.51) as compared to GA (both *P <*0.001).

Binary logistic regression was used, and forward Wald was used to screen independent predictors for the discrimination of GHA from GA. Binary logistic regression analysis revealed that tumor location, PCA, and FAP were independent predictors of GHA discrimination from GA. Using ROC curves, we determined the cut-off value of AFP as 4.7 ng/ml with 79.6% sensitivity and 85.3% specificity with an AUC of 0.881. Similarly, we determined the cut-off value of PCA to be 85 HU with 75.0% sensitivity and 65.5% specificity, with an AUC of 0.749. The combination of tumor location, AFP, and PCA yielded 86.4% sensitivity and 81.9% specificity with an AUC of 0.903. This result is consistent with the findings of Fu et al.; when AFP, CEA, and CT findings were used as criteria, a sensitivity of 97.14% and specificity of 90.91% were achieved (17). However, only 11 patients with GHA were included in this study for analysis.

This study had several limitations. First, three CT scanners were used in our study because of their retrospective nature; however, the CT scanning parameters and contrast media used were similar. Second, some discrepancies during the image analysis were resolved by referral to a senior radiologist. Third, only the CE-CT was investigated in this study. The roles of other imaging techniques, including magnetic resonance imaging (MRI) and positron emission tomography (PET), were not investigated in our study. Further investigations addressing the diagnostic performance of other imaging techniques or comparisons between CT and other imaging techniques are required. Fourth, the number of enrolled GHA patients was relatively small. GHA is an extremely rare malignancy, with published literature limited to case series and observational studies (9, 23). The incidence is difficult to estimate because of the rarity of the disease; one East Asian population study reported an incidence of 0.014 per 100,000 people (24). In the future, we plan to conduct multicenter research to include more patients with GHA to externally validate the diagnostic ability of tumor markers and CE-CT in the discriminative diagnosis of GHA and GA.

In conclusion, AFP levels, tumor location, and CT attenuation values of tumors in the portal venous phase were independent predictors for discriminating GHA from GA. The concentration level of AFP >4.7 ng/ml and CT attenuation values at the portal venous phase <85 HU are indicative of GHA. The combination of AFP, tumor location, and CT attenuation values of tumors at the portal venous phase showed excellent performance in differentiating GHA from GA. This will facilitate better discrimination between these two entities and more closely tailored treatment in cases in which GHA is highly suspected.

## Data availability statement

The raw data supporting the conclusions of this article will be made available by the authors, without undue reservation.

## Ethics statement

The studies involving human participants were reviewed and approved by the ethics committee of Affiliated Hospital 6 of Nantong University. Written informed consent for participation was not required for this study in accordance with the national legislation and the institutional requirements.

## Author contributions

Conceptualization: CD, SR, ZW, and ZD. Data curation: XG, XL, and YW. Formal analysis: CD, and ZD. Resources: XG and YW. Writing—original draft: CD, YW, and SR. Writing—review and editing: SR, ZW and ZD. All authors contributed to the article and approved the submitted version.
